# The Role of Sinusoidal Endothelial Cells in the Axis of Inflammation and Cancer Within the Liver

**DOI:** 10.3389/fphys.2020.00990

**Published:** 2020-08-28

**Authors:** Alex L. Wilkinson, Maria Qurashi, Shishir Shetty

**Affiliations:** Centre for Liver and Gastrointestinal Research, Institute of Immunology and Immunotherapy, University of Birmingham, Birmingham, United Kingdom

**Keywords:** liver sinusoidal endothelial cell, capillarisation, endothelial dysfunction, inflammation, leukocyte recruitment, fibrosis, hepatocellular carcinoma, metastasis

## Abstract

Liver sinusoidal endothelial cells (LSEC) form a unique barrier between the liver sinusoids and the underlying parenchyma, and thus play a crucial role in maintaining metabolic and immune homeostasis, as well as actively contributing to disease pathophysiology. Whilst their endocytic and scavenging function is integral for nutrient exchange and clearance of waste products, their capillarisation and dysfunction precedes fibrogenesis. Furthermore, their ability to promote immune tolerance and recruit distinct immunosuppressive leukocyte subsets can allow persistence of chronic viral infections and facilitate tumour development. In this review, we present the immunological and barrier functions of LSEC, along with their role in orchestrating fibrotic processes which precede tumourigenesis. We also summarise the role of LSEC in modulating the tumour microenvironment, and promoting development of a pre-metastatic niche, which can drive formation of secondary liver tumours. Finally, we summarise closely inter-linked disease pathways which collectively perpetuate pathogenesis, highlighting LSEC as novel targets for therapeutic intervention.

## Introduction

Globally, liver disease is estimated to cause around two million deaths per year, and together, cirrhosis and liver cancer account for 3.5% of all deaths worldwide (Rowe, 2017; Asrani et al., 2019). As the fifth most common cancer and the second leading cause of all cancer-related deaths, hepatocellular carcinoma (HCC) is becoming increasingly prevalent, and is associated with a significant global health burden (Degasperi and Colombo, 2016; Bertuccio et al., 2017; Global Burden of Disease Cancer Collaboration et al., 2017; Asrani et al., 2019). Chronic inflammation plays a critical role in driving the development of HCC, as 90% of patients have an underlying chronic liver disease (CLD) (O’Rourke et al., 2018). Moreover, the liver’s permissiveness to metastasis and the hepatic tropism of many solid cancers makes the liver a frequent site of secondary tumour deposits (Budczies et al., 2015; Mielgo and Schmid, 2020). Since metastasis is responsible for up to 90% of all cancer-related deaths (Chaffer and Weinberg, 2011), better understanding of the factors which permit growth of secondary malignancies is urgently needed. In this review, we will discuss how the unique phenotype and function of liver sinusoidal endothelial cells (LSEC) can contribute in diverse ways to the development of inflammation-induced primary cancer and secondary tumours within the liver.

Liver disease follows a common pathway of progression, from inflammation to fibrosis and cirrhosis, independently of aetiology (Schuppan and Afdhal, 2008; Pellicoro et al., 2014; Koyama and Brenner, 2017). Initial liver injury, which can be toxin-induced, viral, metabolic or autoimmune in origin, causes inflammation which if unresolved can result in chronic hepatitis. Damage to the hepatocytes and changes in the liver microenvironment lead to fibrogenesis (aberrant wound healing and extracellular matrix (ECM) deposition), which can distort the liver architecture and impair liver function and regeneration (cirrhosis). Around 80–90% of HCC cases arise on a background of cirrhosis (Davis et al., 2008; O’Rourke et al., 2018) and, as such, risk factors for HCC development include viral hepatitis, alcoholism and obesity (Degasperi and Colombo, 2016; British Liver Trust, 2019). Many patients are asymptomatic until their liver disease presents at an advanced stage, due to the remarkable compensatory capacity of the liver to fulfill its function even after suffering extensive damage (Schuppan and Afdhal, 2008). Patients with advanced disease have limited therapeutic options and the best outcomes are seen in those patients who are able to undergo liver transplantation. Yet, only 10% of the global transplant demands are fulfilled by current rates (Asrani et al., 2019), thus, there is a vast unmet clinical need to develop novel treatments for liver disease patients.

The liver is strategically positioned to carry out its metabolic and immunological function, since it receives 70–80% of its blood supply from the gastrointestinal tract via the hepatic portal vein, and the remainder from the hepatic artery (Mathew and Venkatesh, 2018). The liver is exposed to countless microbial- and food-derived antigens within the capillary beds of the liver, referred to as the hepatic sinusoids. These vascular beds are lined with specialised discontinuous endothelial cells, known as LSEC, which not only form a unique barrier between the bloodstream and the parenchyma, but also play an integral role in liver physiology, immunology and pathophysiology (Shetty et al., 2018).

An emerging role for LSEC in the development and progression of both CLD and HCC has become evident over the past few decades (Elvevold et al., 2008b; Sorensen et al., 2015; Marrone et al., 2016; DeLeve and Maretti-Mira, 2017; Natarajan et al., 2017; Poisson et al., 2017; Shetty et al., 2018; Hammoutene and Rautou, 2019). Furthermore, the frequent growth of secondary tumours within the liver often requires interactions with LSEC, contributing to a metastatic niche which is exploited by numerous other cancer types (Mielgo and Schmid, 2020). We discuss the role of LSEC in disease pathogenesis and tumour development, highlighting the potential for these cells to be targeted in novel therapeutic approaches.

## LSEC Physiology

### Structure and Location

**FIGURE 1:**
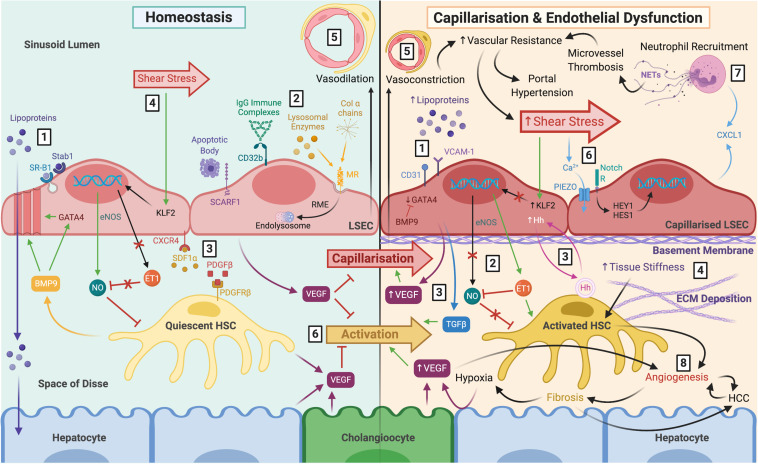
The role of LSEC in maintaining homeostasis and disease pathology following capillarisation and endothelial dysfunction. *Left:* LSEC have a distinct morphology which facilitates their homeostatic function. **(1)** Lack of basement membrane and fenestrations arranged in sieve plates permit relatively free movement of macromolecules, such as lipoproteins, towards hepatocytes within the parenchyma. Lipoproteins can also be endocytosed by scavenger receptors SR-B1 and Stab1. **(2)** Scavenger receptors also facilitate uptake and clearance of waste products including apoptotic cell bodies (SCARF1), IgG immune complexes (CD32b), lysosomal enzymes (MR), and collagen α chains (MR). **(3)** LSEC remain in close proximity with HSC within the space of Disse via CXCR4-SDF1α and PDGF-β PDGFR-β interactions. **(4)** LSEC maintain HSC quiescence in response to shear stress through eNOS-dependent NO production, and inhibition of ET-1, via transcription factor KFL2. **(5)** The differentiated LSEC phenotype maintains vasodilation of the sinusoids. **(6)** VEGF production by LSEC, HSC, hepatocytes and cholangiocytes also maintain HSC quiescence and prevent LSEC capillarisation. *Right:* **(1)** Capillarisation is associated with upregulation of VCAM-1 and CD31, loss of GATA4 signalling, reduced fenestrations, and basement membrane synthesis, leading to hyperlipoproteinaemia. This can be prevented by BMP9. **(2)** Endothelial dysfunction is the inability to produce NO in response to shear stress, and paired with ET-1 synthesis, results in HSC activation. **(3)** Additional angiocrine signals release from capillarised LSEC also perpetuate HSC activation, such as excess VEGF, Hh signals and TGFβ **(4)** Activated HSC begin to deposit ECM which increases tissue stiffness, further stimulating HSC activation. **(5)** HSC respond by causing vasoconstriction which increases vascular resistance and shear stress. **(6)** It is thought that LSEC respond to these mechanocrine signals via PIEZO channels, notch-dependent HEY1 and HES1 translocation and subsequent CXCL1 secretion. **(7)** This leads to neutrophil recruitment, and NETosis induces microvessel thrombosis which perpetuates increased vascular resistance resulting in portal hypertension. **(8)** Ultimately, capillarisation and endothelial dysfunction precede angiogenesis and fibrosis, which increase the risk of cirrhosis and HCC. LSEC, liver sinusoidal endothelial cells; SR-B1, scavenger receptor class B type 1; Stab1, stabilin-1; SCARF1, scavenger receptor class F member 1; CD32b, Fcγ receptor 2b; MR, mannose receptor; RME, receptor-mediated endocytosis; HSC, hepatic stellate cell; CXCR4, C-X-C chemokine receptor type 4; SDF1α, stromal-derived factor 1α; PDGFβ platelet-derived growth factor β; eNOS, endothelial nitric oxide synthase; NO, nitric oxide; ET-1, endothelin-1; KLF2, Krüppel-like factor 2; VEGF, vascular endothelial growth factor; VCAM-1, vascular cell adhesion molecule 1; BMP9, bone morphogenic protein 9; Hh, hedgehog; TGFβ, transforming growth factorβ ECM, extracellular matrix; NETs, neutrophil extracellular traps; HCC, hepatocellular carcinoma.

**FIGURE 2:**
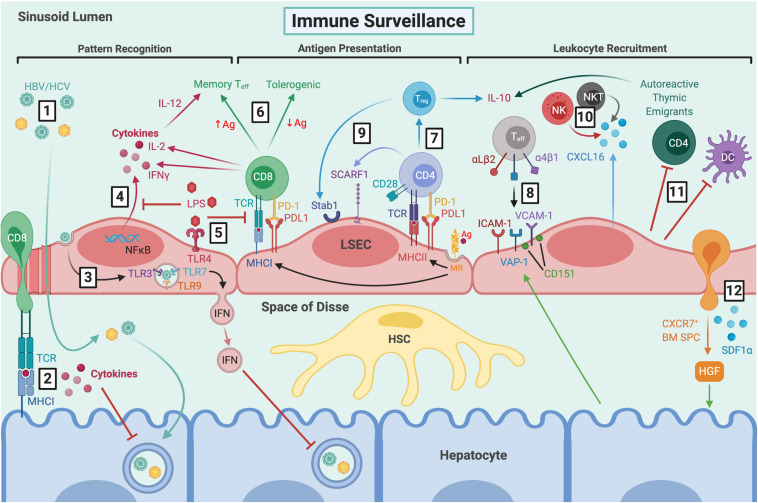
LSEC maintain immune tolerance and facilitate immune surveillance by several mechanisms. **(1)** Viral particles gain access to the parenchyma through fenestrations and HBV/HCV can then go on to infect hepatocytes. **(2)** CD8^+^ T cells extend protrusions through fenestrations and can probe for viral antigens presented by infected hepatocytes via MHCI. **(3)** LSEC can also take up viral particles via transcytosis, and RNA sensing by intracellular TLRs leads to production of IFN-rich exosomes which inhibit viral replication. **(4)** LSEC express pathogen recognition receptors, including TLR4 and NOD1/2, which signal via NFκB leading to cytokine production. **(5)** Clearance of dietary LPS via TLR4 induces tolerogenic responses by inhibiting NFκB translocation and antigen presentation. **(6)** MR-mediated antigen uptake and presentation by MHCI induces tolerogenic CD8^+^ T cell responses in the presence of PDL1, which can be overcome by excess TCR signalling in response to high antigen concentrations. **(7)** MR-mediated uptake also precedes antigen presentation to CD4^+^ T cells via MHCII, leading to T_reg_ induction in the presence of PDL1 and absence of co-stimulation. **(8)** Typically, classic adhesion molecules VCAM-1 and ICAM-1, as well as VAP-1, are involved in T_eff_ recruitment. VCAM-1 is often arranged in microdomains, forming endothelial adhesive platforms in associated with tetraspanin CD151. Hepatocytes can mediate leukocyte recruitment indirectly by modulating expression of adhesion molecules. LSEC are also involved in recruitment of distinct leukocyte subsets via atypical adhesion molecules and chemokines, such as **(9)** CD4^+^ and T_reg_ cells by SCARF1 and Stab1, respectively, and **(10)** production of CXCL16 which promotes retention of CXCR6^+^ NK and NKT cells. **(11)** LSEC also contribute to immune tolerance by inhibiting DCs and promoting apoptosis of CD4^+^ autoreactive thymic emigrants. **(12)** LSEC recruit CXCR7^+^ BM SPCs via SDF1α, which mediate hepatocyte proliferation via HGF production and thus, liver regeneration. LSEC, liver sinusoidal endothelial cells; HBV, hepatitis B virus; HCV, hepatitis C virus; TCR, T cell receptor; MHCI, major histocompatibility complex class I; TLR, toll-like receptor; IFN, interferon; NOD, nucleotide-binding oligomerisation domain; NFκB, nuclear factor κ-light-chain-enhancer of activated B cells; LPS, lipopolysaccharide; MR, mannose receptor; Ag, antigen; PD-1, programmed cell death protein 1; PDL1, programmed death ligand 1; T_eff_, effector T cell; IL-2, interleukin-2; MHCII, major histocompatibility complex class II; T_reg_, regulatory T cell; VCAM-1, vascular cell adhesion molecule 1; ICAM-1, intercellular adhesion molecule-1; VAP-1, vascular adhesion protein 1; Stab1, stabilin-1; SCARF1, scavenger receptor class F member 1; CXCL16, C-X-C chemokine ligand 16; CXCR6, C-X-C chemokine receptor 6; NK, natural killer; NKT natural killer T; DC, dendritic cell; BM SPCs, bone marrow sinusoidal precursor cells; SDF1α, stromal-derived factor 1α; HGF, hepatocyte growth factor; HSC, hepatic stellate cell.

LSEC are the most abundant non-parenchymal cell type in the liver, representing approximately 15–20% of liver cells but only 3% of the total liver volume (Blouin et al., 1977; Poisson et al., 2017; Shetty et al., 2018). Lying at the interface between the systemic arterial and portal venous blood within the sinusoids and the liver parenchyma, they form a unique barrier between the circulation, and the underlying hepatocytes and hepatic stellate cells (HSC) within the space of Disse (Sorensen et al., 2015; Shetty et al., 2018). LSEC are highly specialised in that they have minimal basement membrane and fenestrations, arranged in sieve plates, rendering LSEC the most permeable endothelial cells in the mammalian body (Braet and Wisse, 2002; Poisson et al., 2017). Furthermore, LSEC are ideally positioned to process and recycle blood-borne proteins and lipids both from the gastrointestinal tract and the systemic circulation, thus representing the most powerful scavenger system in the body (Shetty et al., 2018). This is supported by the plethora of endocytic and scavenger receptors expressed in LSEC (Sorensen et al., 2015). Moreover, the atypical cell junctions between LSEC and the low shear environment within the hepatic sinusoids results in differences in leukocyte trafficking compared to the conventional leukocyte adhesion cascade, which may yield specific targets for recruitment during liver disease (Shetty et al., 2008; Patten et al., 2019). It is these key features which distinguish LSEC from other endothelia, allowing them to carry out their homeostatic, filtration, endocytic (Figure 1) and immunological functions (Figure 2).

### Regulation of Hepatic Blood Flow

The liver sinusoids are characterised by low shear flow compared to other capillary beds to maximise time for fluid and solute exchange to occur (Poisson et al., 2017; Mathew and Venkatesh, 2018). LSEC are key regulators of hepatic vascular blood pressure via production of vasodilatory mediators in response to shear stress (Figure 1). This effect is mediated by activation of transcription factor Krüppel-like factor 2 (KFL2), resulting in release of nitric oxide (NO), via endothelial nitric oxide synthase (eNOS) activity in LSEC (Shah et al., 1997; Rockey and Chung, 1998; Parmar et al., 2006; Gracia-Sancho et al., 2011). Simultaneously, shear stress downregulates expression of vasoconstrictive factors, such as endothelin-1 (ET-1), via KLF2 activation (Parmar et al., 2006). These molecules act in a paracrine manner on HSC within the space of Disse, maintaining their quiescent state and thus inhibiting their vasoconstrictive effects (Kawada et al., 1993; Deleve et al., 2008).

Whilst there is some suggestion that LSEC themselves may mediate vascular flow by swelling to form inlet and outlet sphincters (McCuskey, 1966, 2000), the most accepted concept is that LSEC regulate blood flow indirectly via HSC (Rockey, 1997). Hepatic stellate cells are contractile cells, expressing smooth muscle proteins desmin and α smooth muscle actin (αSMA), which enwrap the sinusoids and are ideally positioned to regulate hepatic blood flow (Kawada et al., 1993; Rockey, 1997). As such, LSEC remain in close proximity to HSC via interactions between CXCR4 and CXCL12/stromal-derived factor 1α (SDF1α) released by HSC, along with platelet-derived growth factor β (PDGFβ)-PDGFRβ interactions. Alongside keeping LSEC and HSC in close contact, this cell-cell communication is crucial for vascular tube maturation and integrity during angiogenesis (Hellstrom et al., 1999).

### Barrier Function and Endocytic Properties

The discontinuous nature of the hepatic sinusoidal endothelia permit relatively free trafficking of macromolecules between the blood and the liver parenchyma (Figure 1). The fusion of luminal and abluminal plasma membrane at sites other than cell junctions form fenestrae with diameters of ∼50–150 nm which, unlike other types of fenestrated endothelia, such as those in the pancreas and adrenal glands, lack a diaphragm (Wisse et al., 1985; Stan et al., 2012). Fenestral diaphragms are comprised of plasmalemma vesicle-associated protein (PLVAP) which is the only known molecular component of these structures (Stan et al., 2004, 2012; Ioannidou et al., 2006; Herrnberger et al., 2012). It is thought that PLVAP forms homodimers arranged in radial fibrils, that are anchored to the cell membrane, which regulate vascular permeability by forming a size-selective sieve (Stan, 2004; Rantakari et al., 2015). The role of PLVAP in regulating vascular homeostasis has been recently reviewed (Guo et al., 2016; Bosma et al., 2018).

Despite a lack of PLVAP-containing diaphragms in adult LSEC there is evidence to suggest that PLVAP plays a critical role in development. Namely, it was recently shown that PLVAP regulates the egress of foetal liver monocyte-derived macrophages and subsequent seeding in the tissues, since these cell populations were absent in tissues from *plvap*-deficient mice (Rantakari et al., 2016). Furthermore, PLVAP forms diaphragms in foetal LSEC and is present during foetal angiogenesis in complex with lymphatic vessel endothelial hyaluronan receptor 1 (LYVE-1), neuropilin-1 (NRP-1) and vascular endothelial growth factor receptor 2 (VEGFR2) (Rantakari et al., 2016; Auvinen et al., 2019). Interestingly, Auvinen et al. (2019) also demonstrated PLVAP expression in adult LSEC, which is the first report that clearly defines the expression of PLVAP independently of fenestral diaphragms.

Early structural differentiation of the hepatic sinusoids occurs in human embryos between 5 and 12 weeks of gestation, during which time they downregulate continuous endothelial markers CD31 and CD34 and gain sinusoidal markers CD32 and intercellular adhesion molecule 1 (ICAM-1) (Couvelard et al., 1996; Poisson et al., 2017). Transcription factor GATA4 controls the distinct fenestrated phenotype which LSEC acquire by 20 weeks of gestation (Geraud et al., 2017). Under steady state conditions, fenestrated LSEC allow the passage of metabolites, plasma proteins, lipoproteins and small chylomicron remnants which are taken up by hepatocytes and HSC, whilst blood cells including erythrocytes, leukocytes and platelets are retained within the sinusoids (Poisson et al., 2017). The key role for LSEC in lipid transfer is exemplified following loss of fenestrations, where lipid uptake by hepatocytes is impaired and hyperlipoproteinaemia ensues (Clark et al., 1988; Carpenter et al., 2005; Hagberg et al., 2010; Herrnberger et al., 2014). Thus, LSEC play an integral role in fluid and nutrient exchange and metabolic homeostasis (Figure 1). Interestingly, fenestrations can also be observed in tumour vasculature (Hashizume et al., 2000), which may have implications for tumour persistence and progression, as well as cancer cell invasion and metastasis.

Additionally, the expression of numerous endocytic and scavenger receptors by LSEC permit their phenomenal endocytic capacity which ranks the highest of all cells in the human body (Smedsrod et al., 2009; Poisson et al., 2017). Fenestrations are dynamic structures; they are frequently associated with microtubules and actin filaments of the cytoskeleton, as well as caveolae and clathrin-coated pits, which further facilitates endocytic transport of material to and from the parenchyma (Braet et al., 2009; Mönkemöller et al., 2015). The high endocytic and lysosomal activity of LSEC means they are adept scavengers, playing an important role in the clearance of waste products from the circulation (Figure 1). They recognise and internalise extracellular ligands which are trafficked through the endocytic system and degraded. For instance, CD32b is the only Fcγ receptor expressed by LSEC, which mediates the clearance of small circulating IgG immune complexes (Lovdal et al., 2000; Mousavi et al., 2007).

LSEC further contribute to lipid homeostasis via endocytic uptake of high-density lipoproteins (HDLs) and oxidised or acetylated low-density lipoproteins (LDLs), which is mediated by scavenger receptor type B1 (SR-B1) and stabilin-1 (also referred to as common lymphatic endothelial and vascular endothelial receptor 1 (CLEVER-1) or fasciclin EGF-like, laminin-type EGF-like, and link domain-containing scavenger receptor-1 (FEEL-1) and stabilin-2, respectively (Krieger, 1999; Li et al., 2011). Scavenger receptors also play a key role in maintaining glycoprotein homeostasis (Lee et al., 2002), such as clearance of advanced glycation end (AGE) products (Smedsrød et al., 1997; Svistounov and Smedsrod, 2004). One example is mannose receptor (MR), which binds numerous ligands (Martinez-Pomares et al., 2001), including collagen α chains (Malovic et al., 2007), tissue plasminogen activator (Rijken et al., 1990; Martinez-Pomares, 2012) and lysosomal enzymes which are utilised by LSEC (Elvevold et al., 2008a). LSEC are also capable of taking up antigens, via MR-mediated endocytosis and subsequent antigen presentation (Gazi and Martinez-Pomares, 2009; Martinez-Pomares, 2012; Zehner and Burgdorf, 2013), highlighting that scavenger receptors also possess important immunological functions (Figure 2).

## Immunological Role of LSEC

### Recognition of Danger Signals and Antigen Presentation

LSEC play an integral role in both innate and adaptive immunity and contribute to maintenance of immune tolerance within the liver (Figure 2) (Knolle and Wohlleber, 2016; Wohlleber and Knolle, 2016). LSEC are responsible for the recognition and clearance of microbial antigens and, as such, express many pattern recognition receptors (PRRs) in addition to their scavenger receptors. LSEC respond to stimulation of toll-like receptors (TLR) 1-9 (Martin-Armas et al., 2006; Wu et al., 2010), and constitutively express three protein components of the inflammasome (Boaru et al., 2012) and intracellular nucleotide-binding oligomerisation domain (NOD)-like receptors, including NOD1 and NOD2, along with RIPK2 (Huang et al., 2018). Lipopolysaccharide (LPS) is predominantly cleared by the liver, specifically, 75% by LSEC and 25% by Kupffer cells (KC) (Mathison and Ulevitch, 1979; Yao et al., 2016). LSEC recognition of LPS is mediated by TLR4 and CD14, resulting in activation of the myeloid differentiation primary response gene 88 (MyD88) pathway, nuclear translocation of nuclear factor-κB (NF-κB), and production of pro-inflammatory mediators including interleukin-6 (IL-6) and tumour necrosis factor α (TNFα) (Hayashi et al., 2006; Wu et al., 2010; Faure-Dupuy et al., 2018). However, LSEC responsiveness to LPS diminishes following successive exposure, not due to TLR4 downregulation, but rather a reduction in NFκB nuclear translocation (Uhrig et al., 2005). This tolerogenic response reduced subsequent CD54-mediated leukocyte adhesion, and is thought to represent a mechanism by which LSEC prevent liver over-activation and inflammation in response to low-level dietary LPS (Uhrig et al., 2005). Furthermore, recognition of LPS promotes tolerance induction by downregulating antigen-presentation by LSEC and thus, T cell activation (Knolle et al., 1999).

LSEC also participate in viral clearance (Figure 2). It is estimated that around 90% of the viral load during an infection is cleared by LSEC, as has been shown for adenovirus and human immunodeficiency virus (HIV)-like particles, with the latter being cleared by the sinusoids at an astonishing rate of 100 million particles per minute (Ganesan et al., 2011; Mates et al., 2017). Anderson and colleagues determined the rate of clearance in mice following tail vein infusion with viral particles, by periodic sampling of peripheral blood, and analysis of viral load via quantitative PCR or ELISA over 30 min. Fluorescently labeled viral particles were localised predominantly within LSEC, as determined by fluorescent immunohistochemistry of murine livers following viral infusion. A recent study utilised real-time deconvolution microscopy to show that LSEC contribute to uptake and lysosomal degradation of enterobacterial viruses, such as bacteriophage, acting as a primary anti-viral defense mechanism (Oie et al., 2020). This has implications not only for innate immune responses but also may contribute to the low efficiency of phage therapy, since bacteriophages used for gene delivery appear to be rapidly cleared from the circulation. Furthermore, the morphology of LSEC facilitate immune surveillance against hepatotropic viral infections. Specifically, CD8^+^ T cells have been shown to extend protrusions through LSEC fenestrae, probing for viral antigens presented by infected hepatocytes (Warren et al., 2006; Guidotti et al., 2015).

Antigen uptake via LSEC scavenger receptors, followed by antigen presentation, is a key step in promoting T cell tolerance under physiological conditions (Figure 2). Mannose receptor-mediated uptake, processing and presentation of antigen via major histocompatibility complex (MHC) class I on LSEC facilitates antigen cross-presentation to CD8^+^ T cells (Limmer et al., 2000; Burgdorf et al., 2006, 2007), inducing tolerance via upregulation of co-inhibitory molecule programmed cell death ligand 1 (PD-L1) (Diehl et al., 2008). This has been demonstrated for both oral (Limmer et al., 2005) and tumour antigens (Berg et al., 2006; Höchst et al., 2012). LSEC can also present antigen to CD4^+^ T cells via MHC class II, which is constitutively expressed at low levels, but upregulated in response to inflammatory stimuli (Lohse et al., 1996; Knolle et al., 1998). However, the low expression of co-stimulatory molecules in LSEC, particularly in the presence of IL-10, means they are poor stimulators of naïve CD4^+^ T cell activation (Katz et al., 2004), instead inducing development of regulatory T cells (T_reg_) which suppress immune responses (Carambia et al., 2014). Furthermore, LSEC can impair the ability of DCs to induce naïve T cell proliferation *in vitro*, although the mechanism remains unknown (Bertolino, 2008; Schildberg et al., 2008). LSEC also induce tolerance of recent autoreactive CD4^+^ thymic emigrants, who acquire IL-10-producing capacity and undergo higher rates of apoptosis via enhanced FasL and Bim expression (Xu et al., 2016). Together, these findings define a key role for LSEC in maintaining peripheral tolerance (Figure 2).

Alternatively, LSEC can also present antigen to elicit immunogenic T cell responses (Figure 2). For example, LSEC TLR1/2 stimulation with palmitoyl-3-cysteine-serine-lysine-4 (P3C) induced activation of virus-specific CD8^+^ T cells via low level IL-12 secretion in the absence of PD-L1 expression (Liu et al., 2013). Furthermore, stimulation of NOD1, NOD2 and RIPK2 with diaminopimelic acid (DAP) promotes LSEC maturation and HBV-specific T cell activation (Huang et al., 2018). This effect was mediated by NFκB and mitogen-activated protein kinase (MAPK) activation, and subsequent expression of pro-inflammatory chemokines and cytokines, which ultimately primed HBV-stimulated CD8^+^ T cells to increase their interferon γ (IFNγ) and IL-2 production.

Equally, high antigen concentrations are sufficient to shift a tolerogenic response to an immunogenic one via excess T cell receptor (TCR) signalling (Schurich et al., 2010). Cross-talk between helper CD4^+^ and CD8^+^ T cells is mediated by LSEC, involving simultaneous T cell activation, cross-priming, IL-2 release, TCR stimulation and IL-6 signalling, which ultimately enhances LSEC-primed CD8^+^ T cell effector (T_eff_) functions (Böttcher et al., 2014; Wittlich et al., 2017). These findings provide evidence that LSEC can switch their homeostatic tolerogenic phenotype to an immunogenic one, promoting T cell immunity in response to microbial antigens. Understanding how LSEC mediate liver-specific tolerance and immunity will have important implications when attempting to overcome T cell tolerance, such as during chronic viral infection or liver cancer.

### Leukocyte Recruitment

**FIGURE 3:**
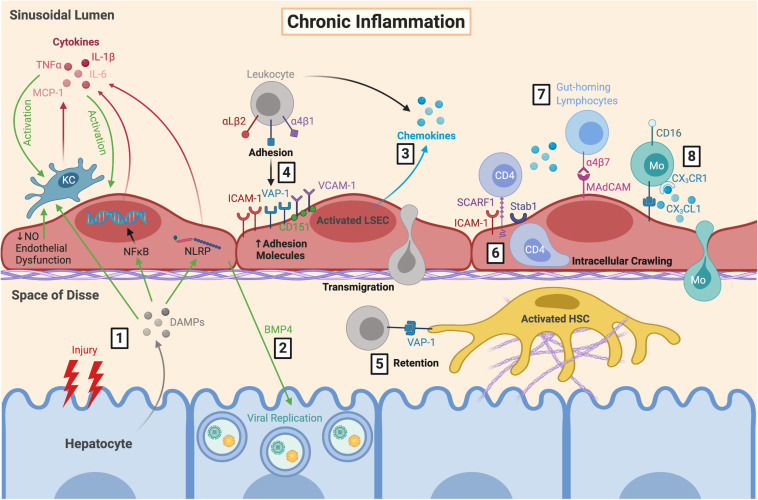
LSEC orchestrate the immune microenvironment during chronic inflammation. **(1)** During chronic inflammation, repeated hepatocyte injury results in release of DAMPs which are sensed by KC, resulting in their activation and subsequent production of pro-inflammatory cytokines. These DAMPs also trigger cytokine release from LSEC via NFκB and inflammasome signalling, further perpetuating LSEC and KC activation. This is exacerbated by endothelial dysfunction. **(2)** Production of BMP4 by LSEC can also promote viral replication which can worsen hepatocyte damage during chronic viral infection. Activated LSEC **(3)** secrete chemokines and **(4)** upregulate their expression of adhesion molecules, which facilitates leukocyte recruitment, adhesion and transmigration. **(5)** Leukocytes can be retained within the space of Disse due to VAP-1 expression by HSC. **(6)** Following SCARF1-mediated adhesion, CD4^+^ T cells have been shown to perform lateral intracellular crawling between LSEC, which is mediated by ICAM-1 and Stab1. LSEC are also important for recruiting distinct pro-inflammatory leukocyte subsets during diseases states, including **(7)** gut-homing lymphocytes via α4β7-MAdCAM interactions, and **(8)** CD16^+^ Mo via secretion of CX_3_CL1. LSEC, liver sinusoidal endothelial cells; DAMPs, danger-associated molecular patterns; KC, Kupffer cell; TNFα, tumour necrosis factor α; MCP-1, monocyte chemoattractant protein 1; IL-6, interleukin-6; NFκB, nuclear factor κ-light-chain-enhancer of activated B cells; NLRP, nucleotide-binding oligomerisation domain, leucine-rich repeat and pyrin domain; NO, nitric oxide; BMP4, bone morphogenic protein 4; ICAM-1, intercellular adhesion molecule-1; VAP-1, vascular adhesion protein 1; VCAM-1, vascular cell adhesion molecule 1; SCARF1, scavenger receptor class F member 1; Stab1, stabilin-1; MAdCAM, mucosal addressin cell adhesion molecule 1; Mo, monocyte; CX_3_CL1, fractalkine; HSC, hepatic stellate cell.

LSEC also contribute to another important aspect of immunity, namely the trafficking of leukocytes through recruitment from the peripheral circulation into the liver (Figure 3). Leukocyte recruitment follows a multi-step adhesion cascade involving several receptor-ligand interactions, which enable capture of circulating immune cells by activated endothelium, and subsequent transmigration to tissue injury or infection sites (Ley et al., 2007). This process is stimulated by recognition of pattern- and danger-associated molecular patterns (PAMPs/DAMPs) by liver immune sentinels, such as KCs, resulting in their activation and subsequent release of cytokines and chemokines. Generally, initial tethering is mediated by selectins and subsequent rolling by integrin activation, which are dictated by the “catch-bond” phenomenon, whereby receptor-ligand interactions are strengthened under conditions of increased shear stress (Marshall et al., 2003; Yago et al., 2007). This brings about cell arrest, followed by intravascular crawling and transmigration through the endothelium into the tissue sites. Typical adhesion molecules involved in leukocyte recruitment include ICAM-1 and vascular cell adhesion molecule 1 (VCAM-1), which form endothelial adhesive platforms by establishing microdomains in association with tetraspanins (Poisson et al., 2017). For instance, CD151 is a tetraspanin which was shown to associate with LSEC VCAM-1 and mediate lymphocyte adhesion under physiological flow conditions *in vitro* (Wadkin et al., 2017).

In contrast to conventional vascular beds, the low shear flow conditions within the hepatic sinusoids leads to lack of selectin-dependent initial tethering and rolling, paving the way for atypical adhesion molecules (i.e., scavenger receptors) to play a more predominant role (Shetty et al., 2008). The involvement of atypical adhesion molecules in leukocyte recruitment has been previously reviewed (Patten and Shetty, 2018). Scavenger receptors are involved in recruitment of distinct leukocyte subsets, and as such, may prove to be novel liver-specific therapeutic targets (Figure 3) (Patten et al., 2019). One example is stabilin-1, which is highly expressed on LSEC in response to hepatocyte growth factor (HGF) and, with the support of adhesion molecules ICAM-1 and vascular adhesion protein 1 (VAP-1), permits the specific transmigration of T_reg_ across the sinusoidal endothelium (Shetty et al., 2011). In addition, scavenger receptor class F, member 1 (SCARF1) mediates selective CD4^+^ T cell adhesion (Patten et al., 2017a), alongside its scavenging functions in the clearance of LDLs and apoptotic bodies (Patten, 2018).

There is also an integral role for chemokines in leukocyte recruitment (Figures 2, 3) (Oo et al., 2010). Chemokines contribute to firm adhesion of leukocytes by binding to G-protein coupled receptors and inducing conformational changes in integrins to facilitate high affinity binding. It is also thought that chemokines are involved in lymphocyte compartmentalisation in liver diseases, with CXCR3 ligands promoting parenchymal recruitment (Curbishley et al., 2005), and CCR5 ligands contributing to recruitment to the portal tracts (Shields et al., 1999; Ajuebor et al., 2004). CXCL9-11 are important for T cell recruitment and transmigration following endothelial activation with IFNγ and TNFα (Curbishley et al., 2005). These chemokine ligands are produced by neighbouring cells and can be transcytosed by LSEC and presented on their cell surface (Middleton et al., 2002; Schrage et al., 2008; Neumann et al., 2015). They can also be circulated within the sinusoids and captured by proteoglycans within the endothelial cell glycocalyx (Curbishley et al., 2005). Fractalkine (CX_3_CL1) interacts with CX_3_CR1 on CD16^+^ monocytes to facilitate adhesion and transmigration in an integrin- and VAP-1-dependent manner (Aspinall et al., 2010). LSEC also express CXCL16 (Geissmann et al., 2005), which interacts with CXCR6^+^ T_eff_ cells to mediate recruitment (Heydtmann et al., 2005; Sato et al., 2005), as well as natural killer (NK) (Hudspeth et al., 2016; Stegmann et al., 2016) and NKT cells (Geissmann et al., 2005) to promote migration during immune surveillance. Thus, chemokines play an important role in recruiting distinct leukocyte subsets across LSEC and maintaining the immune microenvironment within the liver.

Hepatocyte paracrine factors can also enhance expression of LSEC adhesion molecules including ICAM-1, VCAM-1 and VAP-1, indirectly regulating immune cell recruitment (Edwards et al., 2005). LSEC adhesion molecules are relevant in tumourigenesis and metastatic spread and their regulation by transformed hepatocytes or metastatic deposits is of interest. For instance, tetraspanin CD151 is upregulated on LSEC by hepatoma-derived factors and collaborates with VCAM-1 to facilitate recruitment (Wadkin et al., 2017). CD151 has been shown to form endothelial adhesive platforms with VCAM-1 and ICAM-1 in human umbilical vein endothelial cells (HUVEC), permitting lymphocyte adhesion and transmigration, although these structures are yet to be identified in LSEC (Barreiro et al., 2005; Barreiro et al., 2008). Following transmigration, there is also evidence to suggest hepatocytes can modulate T cell populations by engulfment and subsequent lysosomal degradation of autoreactive CD8^+^ T cells (Benseler et al., 2011) and T_reg_ (Davies et al., 2019). These cell-cell interactions have been recently reviewed (Davies et al., 2020).

Although transmigration typically occurs via the paracellular route between endothelial cell junctions, in the liver, lymphocytes frequently extravasate through the endothelial cell body via the transcellular route (Shetty et al., 2011; Patten et al., 2017b). There have also been reports of lymphocyte intracellular crawling within LSEC, shown by live confocal imaging, which was mediated by IFNγ along with ICAM-1 and stabilin-1 expression (Patten et al., 2017b). This demonstrates that LSEC are not just a simple barrier but play an active role in regulating the liver microenvironment. Indeed, the immune microenvironment and leukocyte subsets within it determine the fate of liver injury – resolution, or persistence and chronic hepatitis. Furthermore, excessive immunosuppressive leukocyte subsets promote pathogen escape and tumourigenesis.

## LSEC Pathophysiology

**FIGURE 4:**
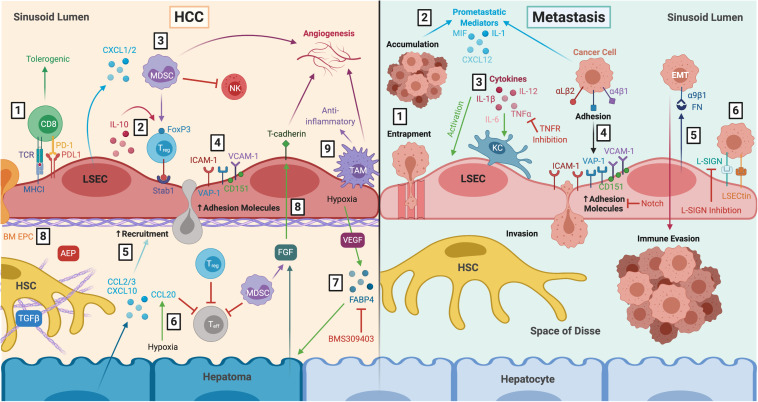
LSEC actively contribute to the tumour microenvironment during HCC and liver metastasis. *Left:* LSEC promote an immunosuppressive microenvironment and thus, HCC development and progression. **(1)** LSEC presentation of tumour antigens to CD8^+^ T cells via MHCI induces tolerogenic responses, in the presence of PDL1, which is upregulated in HCC tumours. **(2)** Production of IL-10 by LSEC induces T_reg_, which are recruited via Stab1, undergoing transmigration and inhibiting T_eff_ responses. **(3)** T_reg_ are also induced by MDSCs, which accumulate in HCC due to LSEC production of CXCL1 and CXCL2. MDSCs elicit pro-tumourigenic effects including inhibition of T cell activation, NK cell inhibition, and stimulation of angiogenesis. **(4)** Transdifferentiated LSEC lose expression of LSEC markers and upregulate expression of adhesion molecules VCAM-1, CD151, VAP-1 and ICAM-1, which facilitates leukocyte recruitment. **(5)** Transformed malignant hepatocytes enhance CCL2, CCL3, and CXCL10 secretion, further promoting leukocyte recruitment. **(6)** Hypoxia-induced production of CCL20 by hepatomas inhibits T cell proliferation. **(7)** LSEC production of adipokines, such as FABP4, in response to hypoxia and VEGF exert pro-oncogenic effects by inducing hepatocyte proliferation. These effects can be attenuated with FABP4-specific inhibitor BMS309403. **(8)** LSEC also foster conditions which promote pro-tumourigenic angiogenesis, including recruitment of pro-angiogenic BM EPC, AEP production and expression of T-cadherin in response to hepatoma- and MDSC-derived FGF. **(9)** Anti-inflammatory TAM also promote immunosuppression and angiogenesis. *Right:* LSEC orchestrate formation of a pre-metastatic niche which promotes development of secondary liver tumours. **(1)** Blood-borne cancer cells can become entrapped within LSEC fenestrae and accumulate in the sinusoidal lumen. **(2)** Tumour cells promote LSEC secretion of pro-metastatic mediators such as MIF, IL-1 and CXCL12, as well as **(3)** activation of KC which produce pro-inflammatory cytokines that in turn activate LSEC. TNFR inhibition has been shown to prevent liver metastasis in mice. **(4)** Activated LSEC upregulate expression of adhesion molecules which promotes binding and invasion of cancer cells to the space of Disse, where they are generally protected from KC and NK cells within the sinusoids. Wnt-independent Notch activation has been shown to inhibit tumour cell adhesion. **(5)** LSEC secrete FN which interact with α9β1 integrin on cancer cells, initiating EMT and promoting metastatic spread. **(6)** LSEC also express L-SIGN and LSECtin, which are upregulated in liver metastasis and mediate adhesion and migration of cancer cells. L-SIGN blockade reduces colon cancer metastasis in murine models. LSEC, liver sinusoidal endothelial cells; HCC, hepatocellular carcinoma; MHCI, major histocompatibility complex I; PD-1, programmed cell death protein 1; PDL1, programmed death ligand 1; IL-10, interleukin 10; T_reg_, regulatory T cell; Stab1, stabilin-1; T_eff_, effector T cell; MDSC, myeloid-derived suppressor cell; CXCL1, C-X-C chemokine ligand type 1; NK, natural killer cell; VCAM-1, vascular cell adhesion molecule 1; VAP-1, vascular adhesion protein 1; ICAM-1, intercellular adhesion molecule 1; CCL2, C-C chemokine ligand type 2; FABP4, fatty acid binding protein 4; VEGF, vascular endothelial growth factor; BM EPC, bone marrow erythroid progenitor cells; AEP, asparaginyl endopeptidase; FGF, fibroblast growth factor; TAM, tumour-associated macrophage; HSC, hepatic stellate cell; TGFβ, transforming growth factor β; MIF, macrophage migration inhibitory factor; KC, Kupffer cell; TNFα, tumour necrosis factor α; TNFR, tumour necrosis factor receptor; FN, fibronectin; EMT, epithelial-to-mesenchymal transition; L-SIGN, lymph node-specific ICAM-3 grabbing non-integrin; LSECtin, lymph node sinusoidal endothelial cell C-type lectin.

**FIGURE 5:**
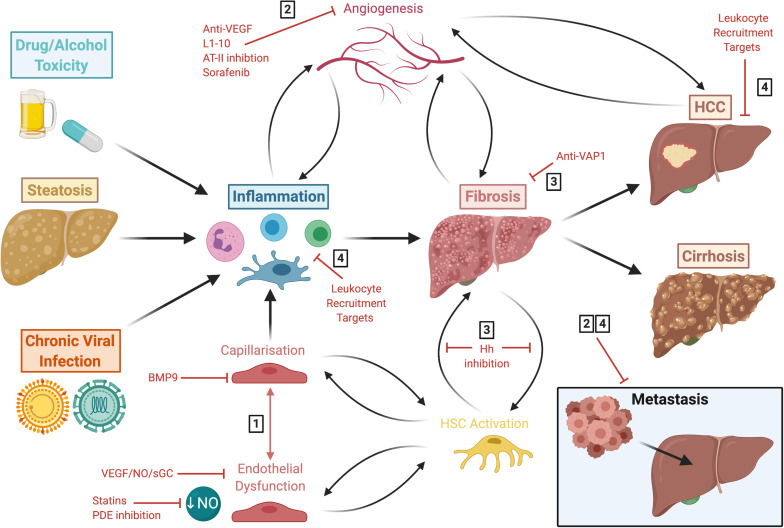
Liver disease follows a common pathway of progression which results in fibrogenesis that is both preceded and driven by LSEC capillarisation and dysfunction. This figure summarises the common disease pathways discussed in this review, highlighting various approaches for potential therapeutic intervention. These include: **(1)** molecules which maintain LSEC homeostasis, such as BMP9, statins and phosphodiesterase inhibitors; **(2)** anti-angiogenics which are also anti-inflammatory and anti-fibrotic, such as L1-10, AT-II inhibition and sorafenib; **(3)** anti-fibrotics, including anti-VAP1 and Hh inhibition; **(4)** and targets involved in leukocyte recruitment. LSEC, liver sinusoidal endothelial cells; HCC, hepatocellular carcinoma; BMP9, bone morphogenic protein 9; VEGF, vascular endothelial growth factor; NO, nitric oxide; sGC, soluble guanylate cyclase; PDE, phosphodiesterase; AT-II, angiotensin II; VAP-1, vascular adhesion protein 1; Hh, hedgehog; HSC, hepatic stellate cell.

An expanding body of evidence strongly implicates LSEC in the development of CLD, and thus liver cancer (Figure 4), due to the nature of liver disease progression from fibrosis to cirrhosis and HCC development (Figure 5). General disease pathways involve chronic liver injury and subsequent endothelial and epithelial damage and dysfunction, which leads to HSC activation, excess ECM deposition, and fibrogenesis. This perpetuates liver damage and can lead to cirrhosis if unresolved (Patten et al., 2019). LSEC contribute to pathogenesis in several ways, by fostering conditions which allow persistence of chronic viral infections and driving processes which initiate and exacerbate fibrosis. These include LSEC capillarisation, characterised by loss of fenestrated morphology and acquisition of vascular phenotype, angiogenesis, and endothelial-to-mesenchymal transition (EndMT). LSEC also release endothelial-derived growth factors, known as angiocrine factors, which determine the balance between regeneration and fibrosis as well as orchestrating tumourigenesis.

### Chronic Viral Infection

As discussed above, LSEC play an important role in viral clearance (Figure 2), by direct sensing of viral RNA which can lead to release of type I and III interferon-rich exosomes and inhibition of viral replication (Pohlmann et al., 2003; Broering et al., 2008; Giugliano et al., 2015). LSEC also recruit and position T_eff_ through expression of ICAM-1, VCAM-1 and VAP-1, and presentation of CXCR3 ligands (Curbishley et al., 2005). They also aid retention of CXCR6^+^ T cells via CXCL16 expression (Heydtmann et al., 2005; Sato et al., 2005).

Conversely, LSEC can also promote hepatotropism of HBV and HCV by permitting them access to the parenchyma, through fenestrations and by transcytosis (Breiner et al., 2001; Protzer et al., 2012). Hence, tolerogenic responses may help viral evasion from the immune system, and LSEC may act as a reservoir for endogenous re-infection. Furthermore, paracrine signals released from LSEC can facilitate HCV replication. One example is bone morphogenic protein 4 (BMP4), whose expression is low in normal liver since it is negatively regulated by VEGFR2 activation, but is upregulated in CLD due to reduced VEGFR2/p38 MAPK signalling (Rowe et al., 2014). LSEC therefore have a pleiotropic role in viral infection, persistence and clearance (Figure 2).

### Sinusoidal Capillarisation and Endothelial Dysfunction

Maintenance of the LSEC phenotype is crucial for them to carry out their physiological function and maintain homeostasis (Figure 1), yet a common response to chronic injury is the development of capillarisation. Sinusoidal capillarisation is the process by which LSEC lose their fenestrated morphology and adopt a more “capillary-like” phenotype. Capillarisation is associated with basement membrane synthesis, loss of GATA4-dependent signals and upregulation of CD31 and VCAM1 (Xu et al., 2003; Shetty et al., 2018). Following production of substantial basement membrane, the phenotypic alterations in LSEC become virtually irreversible. Capillarisation is analogous with endothelial dysfunction, in which LSEC can no longer maintain HSC quiescence in response to shear stress signals (Deleve et al., 2008; Xie et al., 2012), and together these processes precede fibrosis (Horn et al., 1987; Fraser et al., 1991; Pasarín et al., 2012; Baiocchini et al., 2019). A recent study, however, showed that LSEC dysfunction and loss of fenestrations following chronic metabolic stress do not always go hand in hand (Kus et al., 2019). Specifically, it was shown that mice subject to high-fat diet developed non-alcoholic fatty liver disease (NAFLD)-like disease characterised by steatosis, weight gain, insulin resistance and a pro-inflammatory LSEC phenotype, yet LSEC bioenergetics and fenestrae were functionally preserved. This demonstrates the discernible ability of LSEC to adapt to metabolic stress and pro-inflammatory burden associated with NAFLD. Nevertheless, there is compelling evidence to suggest that capillarisation and endothelial dysfunction not only precede fibrosis, but also promote it (Figure 5).

It is well-documented that fenestrations are altered in pathophysiological conditions (Horn et al., 1987; Clark et al., 1988; Fraser et al., 1991; Xu et al., 2003; Baiocchini et al., 2019). Fenestrations also decrease with age (Ito et al., 2007), a process dependent on p53 and p19^*ARF*^- dependent signalling (Koudelkova et al., 2015) which is associated with pseudocapillarisation, sinusoidal dysfunction, loss of vasodilatory capacity, and increased hepatic vascular resistance (Dg et al., 2007; Jamieson et al., 2007; Maeso-Diaz et al., 2018). As eluded to above, fenestrations are dynamic structures which are regulated by several factors and pathways. Studies have shown that cross-talk between LSEC and other hepatic cells can result in loss of fenestrations, for example, LSEC-KC interactions were shown to elicit fenestration loss and upregulation of CD31 (Ford et al., 2015). Additionally, a recent study implicated BMP9, a paracrine factor produced by HSC, as a key regulator of LSEC fenestrations (Desroches-Castan et al., 2019). It was shown that BMP9 maintains vascular quiescence via interactions with its receptor ALK1, and that BMP9 genetic deletion drives LSEC capillarisation and development of perisinusoidal fibrosis in mice. Moreover, addition of exogenous BMP9 to LSEC primary cultures prevented fenestration loss and preserved GATA4 and PLVAP expression.

Behaviour of endothelial cells is regulated largely by mechanical cues of shear stress which is influenced by blood flow through the lumen of the sinusoids (Figure 1). Despite low flow rate, shear stress is generated due to the narrow diameter of the sinusoids. Increasing levels of shear stress result in NO synthesis by LSEC which in turn act to limit vascular resistance by causing vasodilation. Furthermore, the NO signalling pathway maintains the LSEC differentiated phenotype in an autocrine fashion, which is thought to be mediated by the VEGF signalling pathway (DeLeve et al., 2004). Dysfunctional LSEC have impaired eNOS activity, meaning vasodilation switches to vasoconstriction, thus increasing intrahepatic vascular resistance (Rockey and Chung, 1998; Francque et al., 2012). As a compensatory mechanism, LSEC in cirrhotic livers overexpress KLF2 to manage vascular dysfunction, although eventually this is insufficient in preventing portal hypertension and exacerbation of cirrhosis (Gracia-Sancho et al., 2011; Marrone et al., 2013, 2015). Restoration of the NO-dependent pathway via simvastatin (Marrone et al., 2013, 2015; Wang et al., 2013) or sildenafil (Tateya et al., 2011) treatment was shown to improve liver inflammation in rodent steatosis models. Similarly, LSEC differentiation can be re-established by treatment with soluble guanylate cyclase (sGC) activator, BAY-60-2770, which leads to HSC quiescence and attenuation of cirrhosis in rats (Xie et al., 2012).

These pertubations in mechano-sensing by LSEC drive fibrotic processes (Ford et al., 2015; Soydemir et al., 2019) and changes in hepatic blood pressure and liver stiffness occur soon after hepatic injury (Georges et al., 2007). Biophysical characteristics of the ECM and matrix stiffness are key mechanisms in mediating HSC activation contributing to fibrogenesis (Sakata et al., 2004; Olsen et al., 2011). In addition to effects on HSC, mechanical stiffness also impacts hepatocyte phenotype, which is important for regulating cellular responses to tissue injury (Natarajan et al., 2017). Furthermore, mechanical cues are also thought to have indirect effects on LSEC, including cytoskeletal remodelling, loss of fenestrations and formation of stress fibers (Juin et al., 2013; Ford et al., 2015). Hilscher et al. (2019) suggested that activation of PIEZO channels, triggered by integrins and myosin filaments, may be an underlying factor allowing LSEC to respond to changes in shear stress and pressure. Briefly, authors showed that expression of Notch-dependent transcription factors HES1 and HEY1 resulted in neutrophil recruitment via CXCL1 secretion, a mechanism thought to drive microthrombus formation and portal hypertension in mice. Additionally, activated HSC further increase tissue stiffness by depositing more ECM, further driving mechano-activation (Soydemir et al., 2019). Drugs that intervene with this mechano-sensitive positive feedback cycle could show therapeutic promise for the treatment of fibrosis.

Notch signalling has previously been shown to exacerbate LSEC capillarisation via downregulation of the eNOS/sGC pathway (Duan et al., 2018). Notch ligand DLL4 (delta-like ligand 4), which is upregulated in LSEC from cirrhotic patients and carbon tetrachloride (CCl_4_)-treated mice, and has been shown to drive loss of fenestrations and deposition of basement membrane (Chen et al., 2019). The overexpression of DLL4 during liver fibrosis was linked to upregulation of ET-1 and enhanced HSC sinusoidal coverage, which was thought to be initiated by hypoxic conditions associated with fibrogenesis. This validates the Notch pathway and its ligands as potential fibrotic targets, since DLL4 knockdown ameliorated LSEC de-differentiation and provided protection against CCl_4_-induced fibrosis. Contrastingly, Dill et al. (2012) have demonstrated vascular remodelling as a result of disrupted Notch1 signalling, and that maintenance of this signalling pathway conserves the LSEC highly differentiated phenotype. More research is required to fully elucidate the role of Notch signalling in maintenance or alteration of the LSEC phenotype.

An additional pathway that drives LSEC dysfunction is the hedgehog signalling pathway (Xie et al., 2013). Production of hedgehog molecules and other mediators (e.g., transforming growth factor β (TGFβ), laminin and fibronectin) by LSEC activate HSC, which in turn produce hedgehog-containing microparticles that interact with LSEC to further enhance hedgehog signalling (Witek et al., 2009; Xie et al., 2013). Thus, inhibition of hedgehog signalling prevents LSEC capillarisation and restores the differentiated LSEC phenotype (Xie et al., 2013; Zhao et al., 2017). Collectively, these findings exemplify the intimate cross-talk between LSEC and HSC, and the vicious cycle of endothelial dysfunction and HSC activation, which perpetuates fibrogenesis (Figure 5).

### Angiogenesis

The formation of new blood vessels, known as angiogenesis, is a key feature of CLD and HCC that is often associated with areas of fibrogenesis (Coulon et al., 2011; Paternostro et al., 2010). Whilst angiogenesis is a physiological process that is crucial for maintaining homeostasis, in the context of inflammation and endothelial dysfunction, angiogenesis becomes pathological in that it exacerbates fibrotic processes. It is thought that LSEC capillarisation and dysfunction precedes fibrogenesis, that in turn drives angiogenesis, which ultimately perpetuates inflammation and fibrosis (Figure 5) (Kitade et al., 2008, 2009). This is exemplified by the anti-fibrotic action of anti-angiogenic drugs.

Chronic inflammation promotes angiogenesis by several mechanisms, including sustaining hypoxia and inducing transcription of angiogenic hypoxia-inducible factor 1α (HIF1α)-dependent genes and VEGF (Hammoutene and Rautou, 2019). LSEC actively participate in pathological hepatic angiogenesis by releasing pro-angiogenic factors VEGF (Yoshiji et al., 2003), angiopoietins (Taura et al., 2008; Lefere et al., 2019) and adipokines (Kitade et al., 2006) in response to hypoxia, liver injury, inflammation and fibrosis (Zhang et al., 2015). It is known that NRP-1 initiates pro-fibrogenic signalling by promoting HSC activation. A recent study has also implicated NRP-1 in angiogenesis during liver cirrhosis, by upregulation of VEGFR2 expression and activation via PI3K/Akt signalling in LSEC (Wang et al., 2019). Interestingly, NRP-1 and VEGFR2 complex with PLVAP during foetal angiogenesis (Auvinen et al., 2019); whether PLVAP may also drive angiogenesis in adult liver remains to be determined. CD147 has also been shown to promote fibrosis by enhancing hepatic angiogenesis via VEGF-VEGFR2 signalling, which mediated hepatocyte-LSEC cross-talk (Yan et al., 2015). However, when targeting VEGF signalling it should be borne in mind the cell- and context-dependent effects of this approach, since inhibition of VEGFR2 in myeloid cells could both prevent angiogenesis and fibrosis whilst simultaneously hindering LSEC degradation of the ECM and fibrosis resolution (Yang et al., 2014; Kantari-Mimoun et al., 2015).

Angiopoietin-2/Tie2 interactions have been implicated in pathological angiogenesis in non-alcoholic steatohepatitis (NASH), since peptibody L1-10 reduced hepatic angiogenesis and restored normal vascular microarchitecture. In addition, L1-10 treatment downregulated endothelial adhesion molecules VCAM-1, ICAM-1 and monocyte chemoattractant protein 1 (MCP-1), which was also observed in other CLD models including CCl_4_ treatment and bile duct ligation (Lefere et al., 2019). In the same respect, angiotensin II (AT-II) receptor inhibition with candesartan inhibits liver angiogenesis and fibrosis (Yoshiji et al., 2006; Tamaki et al., 2013), whilst anti-VEGFR2 antibodies normalise liver vasculature and reduce inflammatory gene expression in the liver (Coulon et al., 2013). The natural anti-fibrotic compound, Fuzheng Huayu, has been shown to mitigate CCl_4_-induced sinusoidal capillarisation, angiogenesis and expression of angiogenic factors CD31, VEGF, VEGFR2, pERK, and HIF-1α, ultimately reducing liver injury and fibrosis in CCl_4_-treated mice (Liu et al., 2019). Furthermore, targeting of VEGF expression by HSC using compounds such as curcumin (Zhang et al., 2014) and nintedanib (Ozturk Akcora et al., 2017) has also shown to attenuate fibrosis. Inhibition of hedgehog signalling with tetramethylpyrazine reduced angiogenesis and alleviated fibrosis *in vitro* and *in vivo* (Zhao et al., 2017). This was thought to be mediated, at least in part, by restoration of LSEC fenestration and decreased expression of angiogenic markers VEGFA and VEGFR2 as well as endothelial markers CD31 and CD34. These data further support the targeting of angiogenesis to elicit anti-inflammatory and anti-fibrotic effects.

### Angiocrine Factors

Angiocrine factors produced by endothelial cells mediate organ homeostasis, self-renewal and stem cell differentiation, as well as orchestrating tumour growth and metastasis (Figure 4) (DeLeve, 2013). LSEC production of angiocrine signals is tightly regulated and determines the balance between regeneration and fibrosis in response to acute and chronic liver injury. These are mediated by the CXCR7-Id1 and CXCR4 pathways, respectively (Ding et al., 2014). Indeed, poor hepatocyte regeneration correlates with both cellular and functional loss of liver endothelial cells and a decrease in CXCR7-Id1 and HGF expression during acute-on-chronic liver failure (Shubham et al., 2019).

LSEC release HGF and Wnt2 which regulate the functional maintenance and regeneration of hepatocytes. These angiocrine factors are regulated by the VEGF-Id1 axis, since HGF and Wnt2 are not upregulated following partial hepatectomy in Id1 knockout mice (Ding et al., 2010). Further, treatment of LSEC with mitogenic neuropeptide substance P was shown to not only improve endothelial cell viability, proliferation and production of NO/HGF, but also ameliorated TNFα-induced endothelial dysfunction and promoted hepatocyte regeneration (Piao et al., 2019). Interestingly, there is some evidence to suggest that it is not mature LSEC which drive regeneration in the liver, but rather bone marrow-derived progenitor cells of the sinusoidal endothelium. VEGF-SDF1α signalling following liver injury or partial hepatectomy results in recruitment of CXCR7^+^ bone marrow-derived sinusoidal endothelial progenitor cells which mediate liver regeneration (DeLeve et al., 2016).

### Endothelial-to-Mesenchymal Transition

Alongside LSEC phenotypic changes and angiogenesis, there is also evidence to suggest that fibrosis may be driven by endothelial-to-mesenchymal transition (EndMT). The mechanisms by which endothelial cells convert into myofibroblasts is referred to as EndMT, which contributes to ECM deposition and fibrogenesis in liver disease. Healthy LSEC produce modest amounts of collagen type IV and fibronectin under steady state conditions. During liver fibrosis, ECM production increased several-fold but the composition remains relatively consistent (Natarajan et al., 2017). Exposure of LSEC to interstitial collagen fibers and laminin results in defenestration (McGuire et al., 1992; Shakado et al., 1995). Interestingly, culture of LSEC on decellularised liver ECM maintained their fenestrated phenotype for longer periods compared to ECM from other organs (Sellaro et al., 2007).

The production of TGFβ, collagens, fibronectin and laminin by capillarised LSEC may be considered EndMT (Maher and McGuire, 1990; Neubauer et al., 1999), a process characterised by co-expression of CD31 and α-sma (Ribera et al., 2017). EndMT occurs when endothelial cells undergo a series of molecular events and gain a mesenchymal (e.g., myofibroblastic) phenotype, and is a characteristic of many fibrotic diseases (Piera-Velazquez et al., 2016), including cardiovascular and pulmonary disease. However, only a handful of studies have demonstrated EndMT *in vivo* in cirrhotic patients (Dufton et al., 2017; Ribera et al., 2017). These have been validated in CCl_4_ mouse models, where it has been shown that EndMT is mediated by TGFβ – SMAD3 signalling and can be attenuated by BMP7 and etanercept (TNFα inhibitor) treatment (Dufton et al., 2017; Ribera et al., 2017). Decreased EndMT in response to BMP7 treatment correlated with attenuated fibrosis and improved vascular disorganisation (Ribera et al., 2017).

Li Z. et al. (2019) recently showed that inhibition, or endothelial-specific deletion, of transcriptional modulator MKL1 suppresses TGFβ-induced EndMT and associated fibrosis. The driving effect of MKL1 on EndMT was shown to be mediated by recruitment to the promoter region of TWIST1, activating its transcription in a STAT3-dependent manner. Both STAT3 and TWIST1 inhibition, with C188-9 and harmine, respectively, reversed EndMT and bile duct ligation-induced fibrosis in mice, proposing the STAT3-MKL1-TWIST1 axis as a novel fibrogenic pathway with potential for therapeutic targeting. In contrast, endothelial transcription factor (ETS)-related gene (ERG) protects against EndMT by preferentially driving SMAD1 signalling and repressing SMAD3 activity, whilst ERG genetic ablation drove EndMT and spontaneous liver fibrosis (Dufton et al., 2017). Furthermore, decreased ERG expression correlated with EndMT in end-stage liver disease patients (Dufton et al., 2017), suggesting it could be a valid biomarker for assessing EndMT in liver disease.

Clinical studies and animal models suggest that fibrosis can be reversible (Soyer et al., 1976; Hammel et al., 2001; Arthur, 2002; Dixon et al., 2004; Issa et al., 2004). One therapeutic approach is aimed at degradation of the ECM through targeting of matrix metalloproteinases (MMPs) and tissue inhibitors of metalloproteinases (TIMPs) (Clutterbuck et al., 2009; Roderfeld, 2018). Chronic hepatitis patients have lower circulating levels of collagenase, MMP-1, and excess TIMP-1 which positively correlates with aminotransferase levels and fibrosis score (Ninomiya et al., 2001; Flisiak et al., 2004; Guido et al., 2006). Increasing the MMP-1/TIMP-1 ratio has shown promise in chronic hepatitis B and C patients (Ninomiya et al., 2001; Flisiak et al., 2004; Guido et al., 2006). Further, studies in rats have shown that transient overexpression of MMP-1 decreases type I collagen, induces hepatocyte proliferation, and attenuates fibrosis (Iimuro et al., 2003), whilst anti-TIMP-1 antibody treatment reduced collagen accumulation and α-sma expression (Parsons et al., 2004). This is interesting as LSEC, along with HSC, possess MRs which can endocytose denatured collagen α chains from the circulation and space of Disse (Malovic et al., 2007; Madsen et al., 2012). However, MMPs are also implicated in fibrogenesis; their pleiotropic roles in fibrosis have been systematically reviewed (Hemmann et al., 2007). For instance, in mice, MMP-9 has been implicated in activation of latent TGFβ, a process which can drive HSC activation and subsequent collagen deposition, thereby promoting fibrosis (Yu and Stamenkovic, 2000). Nevertheless, it seems that LSEC which have undergone EndMT contribute to fibrogenesis, whilst LSEC which maintain their differentiated phenotype contribute to fibrinolysis and resolution.

### Autophagy

The process of EndMT and TGFβ signalling have been linked to autophagy which is the process of regulated degradation and recycling of intracellular components. Upregulation of TGFβ signalling and EndMT can be induced by loss of autophagy genes, *ATG7* and *ATG4B*, which has been shown to exacerbate inflammation and fibrosis in murine models of cardiac (Li et al., 2016), pulmonary (Cabrera et al., 2015; Singh et al., 2015), renal (Nam et al., 2019a, b), and pancreatic (Zhou et al., 2017) fibrosis. Conflictingly, autophagy has also been defined as a driver of fibrotic disease (Hernandez-Gea et al., 2012; Ghavami et al., 2015; Livingston et al., 2016). Ruart et al. (2019) investigated the role of autophagy in regulating endothelial dysfunction during liver injury. The authors showed that LSEC homeostasis is maintained by autophagy, a process which protects against oxidative stress, that is rapidly upregulated following capillarisation *in vitro* and *in vivo*. Selective loss of endothelial autophagy resulted in reduced intrahepatic NO and subsequent cellular dysfunction which perpetuated fibrosis in CCl_4_-treated mice.

In keeping with these findings, defective autophagy pathways in LSEC have been shown to occur in NASH patients compared with controls and steatotic patients. Autophagy deficiency was linked to liver inflammation, EndMT, apoptosis and perisinusoidal fibrosis in a mouse model of NAFLD, outcomes that were independent of metabolic risk factors such as body weight and plasma cholesterol (Hammoutene et al., 2020). Furthermore, activation of autophagy with hypercholesterolaemia drug ezetimibe, via AMPK activation and nuclear translocation of transcription factor TFEB, ameliorated steatohepatitis by dampening inflammasome signalling in macrophages (Kim et al., 2017). In contrast, autophagy in HSC has been shown to release lipids that promote fibrogenesis (Hernandez-Gea et al., 2012). Thus, the role of autophagy in hepatic fibrosis seems to be cell-specific, with LSEC (Ruart et al., 2019), macrophage (Kim et al., 2017), and hepatocyte (Singh et al., 2009) autophagy providing a protective role, and autophagy in HSC proving detrimental (Hernandez-Gea et al., 2012). This should be considered when designing compounds which target autophagy for fibrotic indications.

## LSEC in the Tumour Microenvironment

Both the structure and function of LSEC render them capable of playing an active role in contributing to the tumour microenvironment (TME) and in the development of primary liver cancer (Figure 4). Of note, the liver is a strongly immunosuppressive environment which acts as protection against inflammation derived from gut antigens. However, the same mechanisms that prevent liver inflammation can foster conditions which promote tumour development.

As discussed previously, LSEC are able to cross-present soluble antigen on their MHCI to CD8^+^ T cells. Due to co-inhibitory signalling, CD8^+^ T cells initially activated by LSEC regain a quiescent state in which they are unable to exert cytotoxic effects upon circulating tumour cells or antigens (Diehl et al., 2008). Thus, LSEC induce tolerance of CD8^+^ T cells toward antigens (Limmer et al., 2000). Moreover, Berg et al. (2006) demonstrated that LSEC are able to induce tolerance toward tumour cells, and Höchst et al. (2012) found that LSEC are involved in tolerance toward carcinoembryonic antigen (CEA), which is associated with colorectal carcinoma. Another mechanism by which the liver sinusoids might be pro-tumourigenic is via the TGFβ-dependent induction of T_reg_ (Carambia et al., 2014).

LSEC transdifferentiation and the loss of several LSEC markers are hallmarks of HCC (Figure 4). Lymphocyte recruitment is promoted by the expression of ICAM-1, VAP-1 and CD151 (Wadkin et al., 2017), and T_reg_ are specifically recruited by stabilin-1 (Shetty et al., 2011). Hepatocyte malignant transformation enhances the secretion of chemokines (Yoong et al., 1999) (including CXCL10, CCL2, and CCL3) and adhesion molecule expression (Yoong et al., 1998) (ICAM-1 and VAP-1) by LSEC, which in turn promotes leukocyte recruitment. As such, HCC tumour tissue is characterised by CD8^+^, CD68^+^, and FoxP3^+^ immune cell infiltrate, particularly within the invasive margin. The expression of PD-L1 on LSEC correlated with both the incidence of CD8^+^ T cells and poor survival outcomes (Ihling et al., 2019). This suggests that HCC may evade immune responses via the upregulation of PD-L1 in response to pre-existing cytotoxic T-lymphocyte activity.

The distinction in the TME between primary liver cancer, which usually arises on a background of chronic liver inflammation, and liver metastases, which arise from an otherwise healthy liver, has been recently reviewed (Figure 4) (Eggert and Greten, 2017). In HCC, both the tumour itself and the underlying chronic inflammation contribute to the TME. Hypoxia, for example, an early event in the development of liver cirrhosis, promotes an immunosuppressive microenvironment. This is relevant in tumourigenesis, where hypoxia induces tumour cells to secrete CCL20, which subsequently inhibits T cell proliferation (Ye et al., 2016). A recent study also highlighted the key role that LSEC play in balancing hepatic immune cell populations through chemokine presentation (Ma et al., 2018). In this study the investigator showed that LSEC presentation of the chemokine CXCL16 was critical in regulating the anti-tumour response of NKT cells in models of primary and metastatic liver cancer.

One of the earliest mechanisms by which the liver is primed for metastasis is by the accumulation of myeloid cells and myeloid-derived suppressor cells (MDSCs) in the liver (Connolly et al., 2010). MDSCs are immature cells of myeloid origin which have been shown to accumulate in the TME of HCC (Hoechst et al., 2008) and contribute significantly to promoting an immunosuppressive niche (Gabrilovich, 2017). MDSCs exhibit pro-tumoural and pro-metastatic effects via a number of diverse mechanisms, including suppression of T cell activation, inhibition of NK cell activity and induction of T_reg_ (Gabrilovich, 2017; Millrud et al., 2017). MDSCs also promote tumourigenesis by remodelling of the TME and tumour angiogenesis via production of VEGF, basic fibroblast growth factor, Bv8, and MMP9 (Ley et al., 2007; Millrud et al., 2017). Chemokines including CXCL1 and CXCL2 are secreted by LSEC, which are involved in the recruitment of MDSCs to the tumour microenvironment (Brodt, 2016). In HCC, MDSC incidence within tumours directly correlated with disease progression and mortality (Lu et al., 2019).

### Metabolism in HCC

In the last few years there has been major interest in the role of metabolism in regulating immunity. It is now accepted that metabolic pathways shape immune responses rather than having a mere bystander role. In turn, metabolic factors, such as altered adipokine signalling and lipid metabolism observed in NASH-associated cirrhotic patients, are linked to inflammation-induced cancer. For instance, linoleic acid accumulation mediates selective loss of intrahepatic CD4^+^ T cells by inducing oxidative stress, which in turn accelerates HCC growth due to impaired anti-tumour surveillance (Ma et al., 2016). The metabolic role of LSEC in lipid metabolism is also likely to contribute to the development of HCC. For example, fatty acid binding protein 4 (FABP4), produced by LSEC in response to hyperglycaemia, VEGF and hypoxia, is increased in NAFLD and HCC patients (Milner et al., 2009; Laouirem et al., 2019). This adipokine exerts pro-oncogenic effects by inducing hepatocyte proliferation, thus enabling HCC to develop in the NAFLD setting (Laouirem et al., 2019). Whilst metformin treatment reduced FABP4 upregulation *in vitro*, specific FABP4 inhibitor BMS309403 reduced tumour growth *in vivo* in murine xenograft models (Laouirem et al., 2019). Adipokines are also known to induce angiogenesis which promotes HCC growth, further highlighting them as a potential target for HCC treatment on a background of NASH. More work is still required to understand how the well-established metabolic role of LSEC impacts the immune and angiogenic environment that supports tumour development within the liver.

### Angiogenesis in HCC

Angiogenesis is a hallmark of all neoplasia, as it facilitates tumourigenesis and metastasis; liver tumours are known to be especially vascularised. During early HCC, there is a switch in tumour blood supply from the portal vein which supplies dysplastic and regenerative nodules as well as 70% of the blood supply to a healthy liver, to the hepatic artery (Semela and Dufour, 2004). There is a growing body of evidence to suggest that LSEC have a role in angiogenesis in the development these hypervascular tumours. HCC tumour progression is associated with phenotypic changes in peri-tumoural LSEC and increased production of angiogenic factors.

There is a strong correlation between changes in LSEC gene expression and angiogenesis in HCC. Endothelial alterations within and proximal to the tumour are associated with HCC. There is a correlation between HCC and a loss of LSEC markers including stabilin-1, stabilin-2, LYVE-1 and CD32b, whilst increasing expression of integrins and ICAM-1 and capacity for angiogenesis, coagulation and fibrinolysis (Wu et al., 2008). Stabilin-2 is an LSEC marker protein; loss of stabilin-2 expression in the endothelial cells of peri-tumoural tissue conferred a significant overall survival advantage (Géraud et al., 2013). It was suggested that this might be via prevention of vascular remodelling and endothelial cell transdifferentiation. Additionally, asparaginyl endopeptidase (AEP), a molecule known to regulate tumour angiogenesis, is absent or low in normal tissues. However, it has found to be significantly upregulated in solid tumours and surrounding ECM, promoting LSEC angiogenesis *in vitro* (Li N. et al., 2019). Therefore, AEP may represent a novel target for progression of CLD and HCC.

The highly angiogenic nature of HCC is associated with increased classical growth factors such as VEGF and PDGF, whilst classical adhesion molecules such as ICAM-1 and VCAM-1 are preferentially expressed on tumour tissue. It has been shown that bone marrow-derived erythroid progenitor cells (BM-EPCs) play a prominent role in HCC angiogenesis (Zhu et al., 2012). LSEC expression of distinct adhesion molecules and growth factors could drive BM-EPCs recruitment, especially since BM-EPC homing to tumour tissue is thought to be via cellular adhesion molecules ICAM-1, VCAM-1, and VEGF.

Another growth factor, FGF2, secreted by HCC and expressed preferentially in tumour tissue compared to CLD, induces T-cadherin on LSEC. T-cadherin is selectively over-expressed in intra-tumoural capillary endothelial cells in many HCC specimens (Adachi et al., 2006). *In vitro*, T-cadherin was found to influence the invasive potential of HCC (Riou et al., 2006), via binding of adiponectin and activation of NFκB, thus preventing tumour cell apoptosis. This suggests T-cadherin might be a mediator of angiogenesis in HCC. Another suggested mediator of angiogenesis is the leukocyte cell-derived chemotaxin 2 (LECT2)-Tie1 signalling pathway, which possesses pleiotropic effects in HCC. On one hand, loss of LECT2 promotes inflammatory monocyte recruitment, suggesting its activity supports an immunosuppressive TME, whilst on the other it acts as a tumour suppressor by inhibiting vascular invasion (Chen et al., 2014). In the context of CLD, LECT2-Tie1 signalling is an important driver of fibrosis by promoting LSEC capillarisation and inhibiting portal angiogenesis (Xu et al., 2019). Consistent with these findings, serum levels of LECT2 are significantly elevated in cirrhotic and HCC patients (Slowik et al., 2019) and correlate with fibrosis stage (Xu et al., 2019), highlighting its usefulness as a potential biomarker or therapeutic target for these indications (Su and Iwakiri, 2020).

Targeting angiogenesis has shown promise in the management of cancer, and current medical therapies focus on this aspect in the setting of HCC. For many years, sorafenib represented the only medical treatment for HCC and increased median survival in advanced HCC by 3 months (SHARP trial) (Llovet et al., 2008). Sorafenib is a multi-kinase inhibitor which acts on VEGF, PDGF and Raf. After a decade as the only approved oral agent for HCC, a second multi-kinase inhibitor, lenvatinib, has now shown to be non-inferior to sorafenib (Kudo et al., 2018). There have been many pre-clinical studies to assess how combinations of anti-angiogenics and other agents may boost therapeutic efficacy. For example, sorafenib inhibits the formation of pre-neoplastic lesions in rat NAFLD models, and combination with AT-II receptor antagonist losartan also had this effect (Yoshiji et al., 2014). AT-II inhibition reduces HIF-1α activity and expression of VEGF which prevents angiogenesis and HCC development in rats (Yoshiji et al., 2006; Tamaki et al., 2013). Inhibition of angiopoietin-2 with L1-10 peptibody reduced angiogenesis, ameliorated steatohepatitis and prevented NASH-associated HCC progression in mice (Lefere et al., 2019). Furthermore, leptin-mediated angiogenesis is known to regulate HCC development and progression (Kitade et al., 2006), and so targeting this adipokine may represent a potential additional anti-angiogenic approach. A key breakthrough in the rationale of targeting vasculature, such as LSEC, in tumour development has come from studies combining immunotherapy and anti-angiogenics, with major interest in the combination of checkpoint inhibitors and anti-VEGF therapies (Hato et al., 2016).

## LSEC in Liver Metastasis

The liver is also the main site of metastasis from a number of primary tumours, including colon, pancreatic and lung (Budczies et al., 2015; Mielgo and Schmid, 2020). The presence of liver metastases is a poor prognostic marker and therefore, there is an urgent need to understand how liver metastases develop, and subsequently identify potential therapeutic targets.

There are four main stages in the development of liver metastases: (i) the microvascular phase, (ii) the extravascular phase, (iii) the angiogenic phase, and (iv) the growth phase. It has been established that tumours “prime” the sites of metastasis. The role of the TME is well-established in liver metastasis, as is the concept of the pre-metastatic niche, in which the TME creates a prime setting for metastases to seed (Figure 4) (Van den Eynden et al., 2013; Brodt, 2016; Mielgo and Schmid, 2020).

LSEC, KC, and NK cells are the first barrier encountered by circulating metastatic cells, forming a natural defense against seeding blood-borne cancer cells. The physical entrapment of tumour cells in the fenestrae of LSEC leads to mechanical stress and deformation. Larger clumps of tumour cells can become trapped in sinusoidal vessels, resulting in localised ischemia-reperfusion, thus triggering the release of NO (Wang et al., 2000) and reactive oxygen species (ROS) from both LSEC and KC (Yanagida et al., 2006). This subsequently results in widespread apoptosis; it has been suggested that up to 95% of circulating tumour cells which encounter LSEC undergo apoptosis (Vekemans et al., 2004).

However, LSEC represent a double-edged sword in liver metastasis. They possess a number of pro-metastatic qualities. Tumour cells promote KC secretion of pro-inflammatory cytokines including TNFα and IL-1. These cytokines stimulate LSEC to upregulate the expression of cellular adhesion molecules including ICAM-1, VCAM-1, endothelial (E)-selectin, and CD31 (Clark et al., 2016). These cellular adhesion molecules can facilitate cancer cell migration to the space of Disse, where there is relative protection from NK and KC (Glinskii et al., 2005). Specifically, cellular adhesion molecules enable tumour cell attachment to LSEC, which in turn promotes tumour cell arrest, extravasation and ultimately, the development of metastasis (Auguste et al., 2007). The proposed mechanisms by which these events occur are detailed below.

### The Role of ICAM-1

Metastatic growth is dependent on tumour-host cell interactions and LSEC are known as key players in this cross-talk. One adhesion molecule shown to be involved in this cross-talk is ICAM-1, which is the main cellular adhesion molecule expressed on LSEC, KC, HSC, and hepatocytes (Zhu and Gong, 2013). ICAM-1 is upregulated by pro-inflammatory cytokines including TNFα, IL-1β, and IFN-γ.

ICAM-1 is a key player in the metastatic process in a number of organs, including lung, liver and blood (Christiansen et al., 1996; Kotteas et al., 2013). It is involved in the first stage of metastasis: adhesion of tumour cells to the endothelial cell wall (in the case of the liver, adhesion of tumour cells to the surface of LSEC). Its expression promotes the activation of pro-metastatic signalling pathways involving IL-6 and IL-8. These pro-inflammatory cytokines increase vascular permeability and further facilitate tumour cell adhesion to the endothelium, thus forming a positive feedback loop promoting tumour seeding and growth. ICAM-1 is also involved in tumour cell extravasation via remodelling of the actin cytoskeleton (Benedicto et al., 2017). *In vivo*, ICAM-1 is implicated in the formation of liver metastases from colorectal cancer (Benedicto et al., 2019), and mediates the infiltration of tumour cells into tumour mass.

### The Role of E-Selectin

The role of sinusoidal E-selectin has been examined by a number of studies. E-selectin mediates the interaction of tumour cells with endothelial cells and is thought to be a critical molecule in tumour adhesion leading to the formation of metastases (Aychek et al., 2008; Tremblay et al., 2008). This correlates clinically; there is increased E-selectin expression found around liver metastases from colorectal primary tumours (Ye et al., 1995). Alexiou et al. (2001) found that elevated levels of E-selectin, ICAM-1 and VCAM-1 correlated with disease outcome in colorectal cancer.

### The Role of Other LSEC-Derived Molecules

It has been shown that LSEC-derived cytokines, including IL-1, macrophage migration inhibitory factor (MIF) and CXCL12 are pro-metastatic (Arteta et al., 2010; Mendt and Cardier, 2017). LSEC secrete fibronectin, which interacts with integrin α9β1 on the surface of colorectal cancer cells, and induces epithelial-to-mesenchymal transition (EMT) via upregulation of Rac signalling pathways and activation of focal adhesion kinase. This mechanism promotes the metastatic capability of colorectal tumour cells (Ou et al., 2014). Kitakata et al. (2002) found that TNFR1 (TNFα receptor)-deficient mice were 50% less likely to develop liver metastases compared to wild-type mice. Wild-type mice injected with tumour cells were found to have significantly increased expression of VCAM-1 and E-selectin on LSEC. Therefore, it is postulated that TNFα plays a key role in the development of liver metastases via its upregulation of VCAM-1 and E-selectin on LSEC.

LSECtin, a C-type lectin, is an adhesion molecule mediating interaction between LSEC and activated T cells. LSECtin is upregulated in liver metastases from colorectal cancer. LSECtin expression correlates with colonic tumour progression and the development of hepatic metastases, which is proposed to be via c-Met upregulation (Zuo et al., 2013). Additionally, lymph node-specific ICAM-3 grabbing non-integrin (L-SIGN/DC-SIGNR/CLEC4M) and LSECtin have been implicated in adhesion and migration of colon cancer cell metastasis to the liver (Liu et al., 2004; Zuo et al., 2013; Na et al., 2017).

## Targeting LSEC in Inflammation and Cancer Therapy

As detailed in the above paragraphs, LSEC are central to co-ordination of the inflammation-cancer axis in the liver (Figure 5). Since alteration in the LSEC phenotype is one of the earliest events in fibrogenesis, and LSEC are known to both perpetuate inflammation and foster the development of primary and secondary liver tumours, they make an attractive target for CLD and oncotherapy. Indeed, there have been recent advancements in this field, notably in approaches aimed at targeting (i) LSEC dysfunction and capillarisation, (ii) angiogenesis and angiocrine signalling, and (iii) leukocyte and/or tumour cell adhesion and transmigration.

Aforementioned approaches which could maintain LSEC differentiation and function include restoration of their fenestrated phenotype, via BMP9 and GATA4 (Geraud et al., 2017; Desroches-Castan et al., 2019), re-constitution of the VEGF/NO/sGC signalling pathway (Tateya et al., 2011; Xie et al., 2012; Marrone et al., 2013, 2015; Wang et al., 2013), and re-establishment of normal hedgehog signalling with hedgehog inhibitors such as tetramethylpyrazine (Zhao et al., 2017). Furthermore, maintenance of autophagy pathways, which are known to be important for endothelial homeostasis and are defective in CLD patients (Hammoutene et al., 2020), may offer an additional treatment strategy. Conservation of the LSEC phenotype and prevention of capillarisation may reduce the risk of progression from inflammation and fibrosis to irreversible cirrhosis and HCC. However, this approach would require timely detection of disease to allow early intervention and prophylaxis. This may be clinically challenging, since liver disease is often initially silent, presenting only in patients with advanced fibrosis. Nevertheless, inhibition of LSEC capillarisation and dysfunction may impede the feed-forward impact on HSC activation, thus promoting fibrosis regression.

Angiogenesis in the context of fibrosis and tumourigenesis has been discussed in this review. The anti-inflammatory and anti-fibrotic effects of anti-angiogenic drugs is well-documented, with some candidates showing promise for CLD and cancer indications. These include peptibody, L1-10, which interferes with angiopoietin-2/Tie2 interactions, AT-II inhibition and multi-kinase inhibitors, sorafenib and lenvatinib, all of which have shown to inhibit both angiogenesis and tumour progression. Other potential targets include AEP and LECT2-Tie1 signalling, although further work is required to validate these candidates.

The recruitment of distinct leukocyte subsets by LSEC presents an opportunity for potential liver- and cell-specific therapeutic targets. Conventional adhesion molecules VCAM-1 and ICAM-1 are known to be upregulated in CLD and cancer, which facilitate leukocyte recruitment. Anti-inflammatory drug, resveratrol, inhibits VCAM-1 expression on tumour-activated LSEC and reduced hepatic melanoma metastases by 75% in a murine model (Salado et al., 2011). Furthermore, ICAM-1 has been demonstrated to play a role in all stages of the metastatic process from adhesion to immune evasion and colonisation. Therefore, ICAM-1 blocking antibodies might represent an avenue of therapeutic interest. To our knowledge, no group has successfully translated ICAM-1 blockade in clinical studies, owing to the challenges associated with inhibition of a cellular adhesion molecule with such important roles in homeostasis (Benedicto et al., 2017).

An alternative approach could be to target atypical adhesion molecules, which are also increased during inflammation and cancer, but display more organ-specific expression. These atypical adhesion molecules include scavenger receptors and VAP-1 which are detailed below. Pre-clinical studies suggest that these endothelial receptors may have roles in recruiting specific subsets of immune cells compared to the more global role of ICAM-1 and VCAM-1. For example, stabilin-1 has been shown to promote T_reg_ recruitment (Shetty et al., 2011) and SCARF1 mediates adhesion of CD4^+^ T cells rather than CD8^+^ T cells (Patten et al., 2017a), under conditions of physiological shear stress, although their specific ligands on T cells are yet to be identified. Endothelial stabilin-1 expression localises to sites of leukocyte recruitment in inflamed liver and is also known to orchestrate B cell recruitment, facilitating adhesion and subsequent intravascular accumulation of B lymphoma cells (Shetty et al., 2012). Targeting of stabilin-1 may therefore allow modulation of the hepatic T_reg_ pool or prevention of tumour metastasis; indeed, genetic deficiency or antibody blockade of stabilin-1 reduces immunosuppressive leukocytes within tumours and halts tumour progression in mice (Karikoski et al., 2014). SCARF1 has been shown to be upregulated in the hepatic sinusoids and at the sites of tissue fibrosis in a range of human CLDs (Patten et al., 2017a). Interestingly SCARF1 is also expressed in well-differentiated HCC tumours, whilst being downregulated in poorly differentiated HCC. VAP-1 was previously shown to promote the recruitment of Th2 lymphocytes rather than the Th1 subset in models of concanavalin A-induced liver injury (Bonder et al., 2005). VAP-1 is highly expressed in CLD (McNab et al., 1996; Weston et al., 2015) and mediates recruitment both directly (Lalor et al., 2002) and indirectly via its enzymatic activity, which can upregulate expression of other adhesion molecules (Lalor et al., 2007; Liaskou et al., 2011). VAP-1 is also expressed by HSCs; blockade of both adhesive and enzymatic properties of VAP-1 attenuates fibrosis, highlighting its therapeutic potential (Weston et al., 2015). Another receptor which has recently been highlighted in single-cell studies of the liver is PLVAP, which is an endothelial-specific protein known to be involved in leukocyte migration across lymphatics (Keuschnigg et al., 2009; Rantakari et al., 2015). Whilst it has an instrumental role in foetal liver-derived immune cell distribution (Rantakari et al., 2016), its involvement in leukocyte recruitment within adult liver remains to be explored. Nevertheless, PLVAP seems to be upregulated during inflammatory states (Ramachandran et al., 2019; Su et al., 2020), and its re-emergence in the HCC microenvironment (Wang et al., 2014) suggests a role in pathogenesis which requires further investigation.

Inhibition of LSEC-cancer cell interactions represents an additional strategy. Blockade of L-SIGN has shown promise in reducing colon cancer metastasis in murine models (Zuo et al., 2013; Na et al., 2017), whilst MR has been suggested as a potential target for treating hepatic metastases and increasing anti-tumour cytotoxicity (Arteta et al., 2010). Moreover, LSEC Notch activation negatively regulates tumour cell adhesion in a Wnt-independent manner, thus protecting against liver metastasis (Wohlfeil et al., 2019). Selective modulation of Notch activity has been proposed as a therapeutic option in liver metastasis. The findings presented here suggest that the targeting of LSEC receptors and LSEC-specific pathways may be an attractive therapeutic strategy to modulate the hepatic immune microenvironment. Which pathway to target will be context-dependent; in CLD, the aim would be to promote anti-inflammatory and pro-resolution conditions, while in HCC, there will also be a need to drive an immunogenic, anti-tumoural environment to support cancer immunotherapy. Furthermore, targeting of cancer cell adhesion to LSEC may offer a route to mitigate frequent metastasis of solid tumours.

It is likely that the success of future LSEC treatments will depend on the reliable delivery of agents to the sinusoidal vascular bed in order to avoid systemic complications. This has led to several approaches to directly target LSEC in pre-clinical models (Poisson et al., 2017). For example, nanoparticles developed to interact exclusively with LSEC have been trialled in murine models of liver metastasis. Yu and colleagues reported that LSEC are specifically targeted by alpha-melittin nanoparticles (Yu et al., 2019), which increase numbers of innate and adaptive immune cells in addition to promoting T cell activation and NK cell maturation. Their ability to prime the anti-tumour response prevents the formation of metastasis. Moreover, melittin nanoparticles have a direct cytotoxic effect on metastatic tumours. Mice treated with melittin nanoparticles had significantly improved survival rates (Yu et al., 2019). Using miRNA conjugated with chondroitin sulfate-functionalised nanoparticles, Marquez and colleagues targeted LSEC activation during angiogenesis. MiR-20a, a molecule known to be downregulated in colorectal liver metastasis, was replaced in LSEC of murine models of liver metastases (Marquez et al., 2018). They found that targeting MiR-20a replacement reduced LSEC recruitment into metastatic foci, and metastasis size decreased by 80% following treatment. The development of nanoparticles specifically targeting LSEC is of particular interest as it enables a narrow therapeutic focus, potentially reducing the risk of systemic toxicity. These approaches will hopefully lead to the successful translation of LSEC-specific agents into clinical trials.

## Conclusion

LSEC play essential roles in physiology and homeostasis, with their dysfunction preceding fibrogenesis that yields high risk of carcinogenesis. Context-dependent LSEC signalling determines the outcome of liver injury, either tissue regeneration or fibrosis, providing strong support for LSEC as a target for fibrotic disease. Furthermore, their active involvement in modulating the immune and tumour microenvironment not only fosters primary and secondary tumour development, but also offers promising opportunities for therapeutic intervention.

## Author Contributions

AW, MQ, and SS wrote the review. AW prepared the figures. All the authors contributed to the article and approved the submitted version.

## Conflict of Interest

SS receives consultancy fees from Faron Pharmaceuticals. The remaining authors declare that the research was conducted in the absence of any commercial or financial relationships that could be construed as a potential conflict of interest.
